# Characterization of a Cancer-Induced Bone Pain Model for Use as a Model of Cancer Cachexia

**DOI:** 10.3390/cimb46120797

**Published:** 2024-11-23

**Authors:** Takuya Hasegawa, Kohichi Kawahara, Koji Sato, Yoshihisa Asano, Takehiko Maeda

**Affiliations:** 1 Department of Pharmacology, Faculty of Pharmacy, Niigata University of Pharmacy and Medical and Life Sciences, 265-1 Higashijima, Akiha-ku, Niigata 956-8603, Japan; p23d01g@st.nupals.ac.jp; 2 Department of Bio-Analytical Chemistry, Faculty of Pharmacy, Niigata University of Pharmacy and Medical and Life Sciences, 265-1 Higashijima, Akiha-ku, Niigata 956-8603, Japan; kkawa@nupals.ac.jp; 3 Laboratory of Health Chemistry, Faculty of Pharmacy, Niigata University of Pharmacy and Medical and Life Sciences, 265-1 Higashijima, Akiha-ku, Niigata 956-8603, Japan; ksato@nupals.ac.jp

**Keywords:** cancer cachexia, animal model, nutritional impact symptoms

## Abstract

Cancer cachexia is a debilitating syndrome characterized by progressive weight loss, muscle wasting, and systemic inflammation. Despite the prevalence and severe consequences of cancer cachexia, effective treatments for this syndrome remain elusive. Therefore, there is a greater need for well-characterized animal models to identify novel therapeutic targets. Certain manifestations of cachexia, such as pain and depression, have been extensively studied using animal models of cancer-induced bone pain (CIBP). In contrast, other aspects of cachexia have received less attention in these models. To address this issue, we established the CIBP model by injecting Lewis lung carcinoma into the intramedullary cavity of the femur, observed cachexia-related symptoms, and demonstrated the utility of this model as a preclinical platform to study cancer cachexia. This model accurately recapitulates key features of cancer cachexia, including weight loss, muscle atrophy, adipose tissue depletion, CIBP, and anxiety. These findings suggest that psychological factors, in addition to physiological and metabolic factors, play significant roles in cancer cachexia development. Our model offers a valuable resource for investigating the underlying mechanisms of cancer cachexia and for developing innovative therapeutic strategies that target physical and psychological components.

## 1. Introduction

Cancer cachexia is a progressive metabolic syndrome characterized by systemic metabolic alterations, involuntary loss of skeletal muscle and adipose tissue, and functional decline of multiple organs [1,2,3,4]. Conventional nutritional support is insufficient to correct these symptoms completely. Cancer cachexia impairs the efficacy of anticancer therapy and radiotherapy and increases the risk of surgical complications and treatment-related toxicity [2,5]. The incidence of cancer cachexia in patients with advanced cancer ranges from 50% to 80% and is associated with mortality in more than 20% of patients with cancer [6,7].

Cancer cachexia results from a complex interplay between various factors, including increased systemic inflammation, elevated energy expenditure, reduced energy intake, enhanced catabolism, and biological pathways [8,9,10,11,12]. However, its exact etiology remains elusive. International guidelines for managing cachexia–anorexia recommend a multidisciplinary approach incorporating nutritional, physiological, and pharmacological interventions to enhance quality of life (QOL) for affected patients [13]. Corticosteroids, nonsteroidal anti-inflammatory drugs, eicosapentaenoic acid, and progesterone-based agents have shown promise in managing cancer cachexia [14,15]. However, their clinical use remains limited. Oral anamorelin, a ghrelin receptor agonist, was approved in Japan in 2021 as a novel therapeutic option for patients with cancer cachexia. This agent stimulates growth hormone secretion by activating the growth hormone secretagogue receptor type 1a (GHS-R1a), thereby increasing appetite [16]. Clinical studies have demonstrated the efficacy of anamorelin in increasing weight and improving quality of life in patients with non-small cell lung cancer. However, although anamorelin increases the lean body mass of patients with cancer cachexia, it fails to improve physical function [17,18,19]. Furthermore, its administration is contraindicated in conjunction with CYP3A4 inhibitors and in patients with hepatic or cardiac impairment [20,21]. Consequently, elucidating the complex etiopathogenesis of cancer cachexia remains imperative to identify novel therapeutic targets and develop effective treatment options.

The development of effective countermeasures for cancer cachexia relies heavily on the use of appropriate animal models. Various such models have been established, with most involving the subcutaneous implantation of tumor cells into rodents. Common tumor cell lines employed include murine colon adenocarcinoma (Colon-26) [22], Lewis lung carcinoma (LLC) [23], and B16F10 melanoma [24], as well as human gastric adenocarcinoma (85As2) [25] and rat hepatocellular carcinoma (Yoshida AH-130) [26]. Additionally, genetically engineered mouse models, such as *Apc* *^Min^*^/^*^+^* [27], inhibin alpha subunit knockout model [28], and KPP (*Ptf1a^Cre-ERTM^; Kras^LSL-G12D^; Pten^flox^*) model [29], have contributed significantly to this field. While animal models have significantly advanced our understanding of cancer cachexia, no single model fully recapitulates its multifaceted nature, including tumor heterogeneity, complex host–tumor interactions, therapeutic interventions, psychological factors, and comorbidities. Therefore, novel preclinical models that recapitulate key clinical features of cancer cachexia must be developed.

Bone metastases are common in prostate, breast, and lung cancer and are found in about 85% of patients dying from these cancers [30]. Therefore, 75–90% of patients with metastatic or advanced cancer experience significant cancer-related pain [31,32]. Pain can occur at any stage of the disease but typically increases with time. It has been suggested that cancer pain is an important predictor of the severity of cachexia and may lead to reduced QOL [33]. Some symptoms of cachexia, such as pain and depression, have been the subject of extensive study in animal models of cancer-induced bone pain (CIBP). However, other aspects of cachexia have only been the focus of limited investigation in these models [34,35].

Therefore, in this study, we created a CIBP model by injecting LLC into the intramedullary cavity of the mouse femur and investigated the characteristics of cachexia in this model. This model recapitulates key features of cancer-associated cachexia, including progressive muscle and adipose tissue depletion, anorexia, cancer-induced bone pain, and anxiety-like behavior. This study aims to characterize this model as a suitable preclinical platform for investigating the pathogenesis of cancer cachexia.

## 2. Materials and Methods

### 2.1. Cell Culture

LLC cells were obtained from AntiCancer Japan and cultured in high-glucose Dulbecco’s Modified Eagle’s Medium (Sigma-Aldrich, Burlington, MA, USA) supplemented with 50 U/mL penicillin, 0.05 mg/mL streptomycin (Thermo Fisher Scientific, Waltham, MA, USA), and 10% fetal bovine serum (FBS; JRH Biosciences, Lenexa, KS, USA). The cells were maintained at 37 °C in a humidified atmosphere containing 5% CO_2_. Cells were passaged every 4 days using 0.25% trypsin/EDTA (Thermo Fisher Scientific) and reseeded onto collagen-coated culture plates (AGC TECHNO GLASS, Haibara, Shizuoka, Japan).

### 2.2. Animals

Male C57BL/6J mice (6–8 weeks old) were obtained from SLC (Hamamatsu, Shizuoka, Japan). The mice were housed in a specific pathogen-free environment at 23 ± 1 °C with a 12 h light/12 h dark cycle and provided with a standard chow diet. One hundred and five C57BL/6 mice were randomly assigned to either a tumor-implanted group (n = 68) or a control group (n = 37) and housed four per cage. All animal procedures were approved by the Animal Care and Use Committee of Niigata University of Pharmacy and Medical and Life Sciences and were conducted in accordance with the institution’s guidelines for the care and use of laboratory animals (permit number 21-7). Euthanasia was performed according to humane endpoints (The experiment was terminated when the animals lost more than 20% of their body weight within a week).

### 2.3. LLC Cell Inoculation

LLC cells were dissociated into a single-cell suspension using 0.25% trypsin/EDTA and resuspended in 5% FBS in phosphate-buffered saline (PBS) at a concentration of 1 × 10^4^ cells/μL. The mice were anesthetized with a combination of ketamine (37.5 mg/kg) and medetomidine (0.25 mg/kg), and a small hole was made in the right femur using a 26-gauge needle. A total of 5 × 10^4^ LLC cells in 5 μL of the cell suspension were injected into the femoral bone marrow cavity using a 26-gauge Hamilton syringe. Sham mice received an equivalent volume of 5% FBS in PBS. Anesthesia was reversed with atipamezole (2.5 mg/kg).

### 2.4. Dissections

Dissections were performed 4 weeks after LLC transplantation. The mice were anesthetized with a combination of ketamine (70 mg/kg) and medetomidine (0.5 mg/kg). Blood samples were collected from the inferior vena cava of the mice. Various tissues, including the tibialis anterior (TA), extensor digitorum longus (EDL), gastrocnemius (GAS), soleus (SOL), epididymal white adipose tissue (eWAT), heart, and liver, were meticulously dissected and then weighed on an electronic balance. Tissue from the tumor-free side was utilized for subsequent analyses.

### 2.5. Quantitative Real-Time Polymerase Chain Reaction (PCR)

Total RNA was extracted using the RNAiso reagent (Takara Bio, Kusatsu, Shiga, Japan), and 500 ng was reverse transcribed to cDNA using the PrimeScript™ RT reagent kit (Perfect Real Time; Takara Bio). Quantitative real-time PCR was conducted using GoTaq^®^ qPCR Master Mix (Promega, Madison, WI, USA) on a MiniOpticon RT-PCR system (Bio-Rad, Hercules, CA, USA). The following cycle parameters were employed: denaturation at 95 °C for 30 s, annealing at 58 °C for 30 s, and elongation at 72 °C for 30 s. Relative expression was calculated with normalization to glyceraldehyde-3-phosphate dehydrogenase (Gapdh) values using the ΔΔCt method. The sense and antisense primers utilized are listed in Table 1.

### 2.6. Skeletal Muscle Histology

TA samples were frozen in isopentane and cooled with liquid nitrogen for later histochemical analyses. Serial sections (10 μm) were cut transversely through the TA muscle using a refrigerated (−20 °C) cryostat microtome (OSK 97LF509; Ogawa Seiki Co., Ltd., Nakaku, Hiroshima, Japan). The sections were stained with hematoxylin and eosin (H&E) to determine the general muscle architecture and measure the mean myofiber cross-sectional area (CSA). Digital images were captured using an Olympus BX43 microscope (Olympus Corporation, Hachioji, Tokyo, Japan) equipped with an Olympus DP74 digital camera system. Image analysis and quantification were performed using ImageJ software (Ver.1.54g). For each frozen sample (n = 6), the CSA of at least 150 skeletal muscle fibers was measured.

### 2.7. Grip Strength Test

Grip strength was assessed using a GPM-101B grip strength meter (Melquest, Toyama, Japan) in accordance with standard protocols [36]. The mice held onto a wire grid, and the highest force exerted during tail traction was measured in grams using a digital force transducer. This measurement represents the maximum grip force exerted by the animal prior to releasing the wire grid. This procedure was repeated five times with a 1 min interval between trials. Data are presented as the mean of five measurements per mouse (n = 5). Analyses were conducted independently by multiple blinded experimenters.

### 2.8. Adipose Tissue Histology

The mice were deeply anesthetized with a combination of ketamine and medetomidine before undergoing intracardiac perfusion with 25 mL of PBS followed by 4% paraformaldehyde in PBS. Epididymal adipose tissue was subsequently removed and post-fixed in 4% paraformaldehyde/PBS for 48 h. Subsequently, the samples were cryoprotected by immersion in a graded sucrose (10%, 20%, and 30%, 24 h each), embedded in OCT compound (Sakura Finetek, Tokyo, Japan), and then flash-frozen in liquid nitrogen. Cryostat sections (10 μm) were cut using an OSK 97LF509 cryostat at −25 °C and then stained with H&E. Images were captured using an upright microscope equipped with a digital camera and analyzed using ImageJ software (Ver.1.54g). The CSA of at least 150 cells was measured for each frozen sample (n = 6).

### 2.9. Determinations of Tumor Necrosis Factor (TNF)-α and Interleukin (IL)-6 Concentrations in Plasma

Blood samples were centrifuged at 2500× *g* for 10 min at 4 °C using heparinized tubes. Isolated plasma was stored at −80 °C until subsequent analyses. Plasma TNF-α and IL-6 levels were quantified using a ProQuantum Immunoassay Kit (Thermo Fisher Scientific) in accordance with the manufacturer’s protocol.

### 2.10. Forced Swim Test (FST)

A cylindrical chamber (30 cm height × 20 cm diameter) was filled with water to a depth of 15 cm and maintained at 25 °C. On the preceding day, the mice were acclimated to the apparatus for 10 min. On the test day, the mice were subjected to a 5 min FST. Immobility time, defined as the duration of floating without active struggling, was recorded as a measure of depressive-like behavior [37]. Analyses were conducted independently by multiple blinded experimenters.

### 2.11. Tail Suspension Test (TST)

The mice were suspended by their tails using adhesive tape attached to a horizontal rod positioned 20 cm above the ground. The duration of the test was 6 min, during which immobility time, defined as the absence of escape-oriented behaviors, was recorded [38]. Analyses were conducted independently by multiple blinded experimenters.

### 2.12. Elevated Plus Maze (EPM) Test

The elevated plus maze (EPM) apparatus consisted of two opposite open arms (50 cm × 12 cm) and two enclosed arms of identical dimensions surrounded by 50 cm high walls. The central platform, facilitating movement between arms, measured 12 cm × 12 cm. The entire maze was elevated 50 cm above the ground. For the EPM test, the mice were individually placed in the central area facing a closed arm and allowed to explore for 5 min. The number of entries into and time spent in open and closed arms were recorded. Reduced time spent and decreased entries into the open arms are indicative of anxiety-like behavior [39,40]. Entry rate in the open arms (%) was determined using the following formula: (open arm crossing/total arm crossing) × 100. Analyses were conducted independently by multiple blinded experimenters.

### 2.13. Weight-Bearing Test

Animals were acclimatized to the testing environment for a minimum of 30 min prior to pain assessment on days 7, 14, and 21 post-inoculation. Hindlimb weight distribution was assessed using an incapacitance meter tester (Model 600M, IITC Life Science, Woodland Hills, CA, USA). The weight borne by each hindlimb was recorded in grams over a 5 s interval [36]. The mean of three measurements was calculated for each animal. The weight-bearing ratio (%) of the weight of the tumor-bearing hindlimb was determined using the following formula: (weight on the tumor-bearing hindlimb/weight on the contralateral hindlimb) × 100. Analyses were conducted independently by multiple blinded experimenters.

### 2.14. Statistical Analysis

Data are presented as mean ± standard error of the mean (SEM). Statistical analysis was performed using Prism 9 (GraphPad Software, Boston, MA, USA). The unpaired Student’s *t*-test and Mann–Whitney U test were used for normally distributed and non-normally distributed data, respectively. Differences were considered statistically significant at * *p* < 0.05, ** *p* < 0.01, *** *p* < 0.001, and **** *p* < 0.0001.

## 3. Results

### 3.1. Changes in Body Weight, Food Intake, and Water Intake of Mice Implanted with LLC Tumors in the Femoral Intramedullary Space

**Figure 1:**
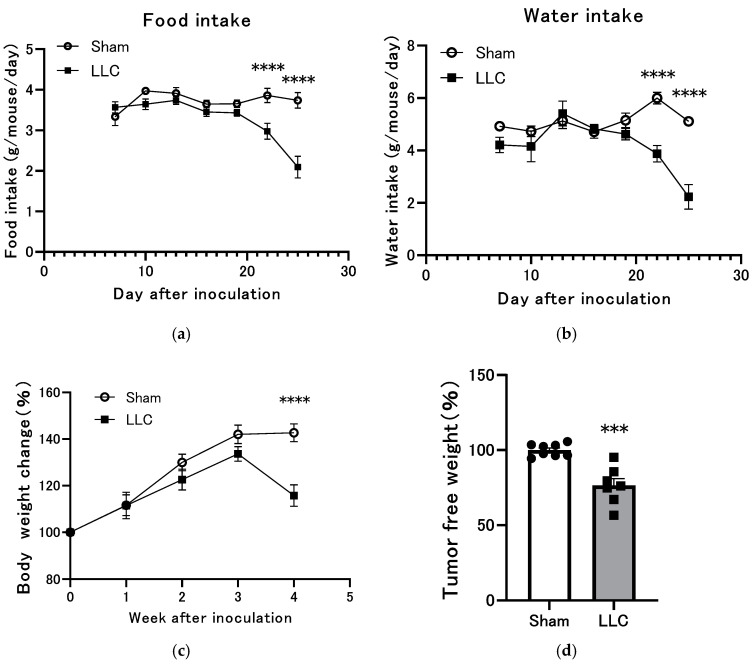
Changes in body weight, food intake, and fluid intake of mouse model with femoral implanted Lewis lung carcinoma (LLC) cells. (**a**) Food intake and (**b**) water intake were recorded every 3 days for 4 weeks. (**c**) Change in body weight per week. (**d**) Final body weight (excluding tumor mass) was calculated on the concluding day of the study. Data are expressed as mean ± SEM (n = 7–8). *** *p* < 0.001, and **** *p* < 0.0001 compared with the control group.

The mice in the LLC group received a transplant of LLC cells into the femoral bone marrow cavity of the right hind limb. To evaluate the development of cachexia, food and water intake were monitored every 3 days. Both food and water intake gradually decreased in the LLC group from day 21 onward (Figure 1a,b). The mice in the LLC group exhibited significantly lower weight gain than those in the sham group from days 21 to 28 (Figure 1c). At the experimental endpoint, the mice in the LLC group displayed significantly lower body weight than those in the control group (Figure 1d). These findings collectively indicate that femoral LLC implantation induces weight loss and anorexia, which are hallmarks of cancer cachexia.

### 3.2. Femoral Implantation of LLC Cells Induces Progressive Skeletal Muscle Atrophy

**Figure 2:**
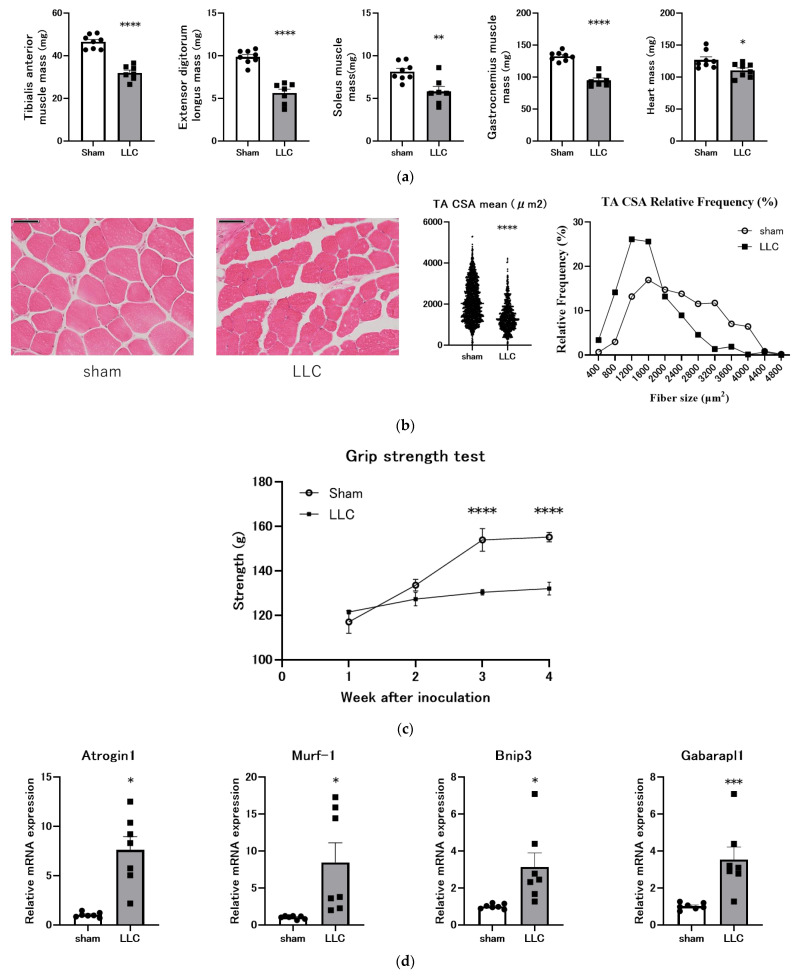
Significant muscle wasting occurs in a cancer cachexia model with Lewis lung carcinoma (LLC) implanted in the femoral cavity. (**a**) Evaluation of relative changes in mass of heart and skeletal muscles (tibialis anterior (TA), extensor digitorum longus (EDL), soleus (SOL), and gastrocnemius (GAS)) 4 weeks after LLC transplantation (n = 7–8). (**b**) Hematoxylin and eosin-stained sections (scale bar, 50 μm) and cross-sectional area (CSA) of TA muscles (n = 6). (**c**) Forearm grip strength (grams) over time. (**d**) Relative mRNA expression levels of *Atrogin-1*, *MuRF-1*, *Bnip3*, and *Gabarapl1* (n = 7). Statistical comparisons were performed using a two-tailed unpaired *t*-test for normally distributed data or Mann–Whitney U test for non-parametric data. Significance levels are indicated as * *p* < 0.05, ** *p* < 0.01, *** *p* < 0.001, and **** *p* < 0.0001.

Compared with the mice in the sham control group, the mice in the LLC group exhibited significantly lower mass of the EDL, TA, GAS, and soleus (Figure 2a). The mice in the LLC group also had significantly lower heart weights than those in the sham control group (Figure 2a). Histological analysis of the TA revealed that the myofiber CSA was significantly smaller in the LLC group than in the sham control group (Figure 2b). Additionally, grip strength was significantly lower in the LLC group than in the sham control group from 3 weeks post-implantation (Figure 2c). The mRNA expression levels of the atrophy-related genes *Atrogin-1* [41], *MuRF-1* [41], *Bnip3* [42,43], and *Gabarapl1* [42,43] were quantified in the TA to explore the potential mechanisms underlying muscle atrophy. The expression of these genes was significantly upregulated in the LLC group compared with the sham control group (Figure 2d). These findings collectively demonstrate that femoral LLC implantation induces skeletal muscle atrophy, characterized by muscle mass loss, myofiber atrophy, and increased expression of atrophy-related genes, which are consistent with the phenotype of cancer cachexia.

### 3.3. Femoral Implantation of LLC Cells Induces Adipose Tissue Loss

**Figure 3:**
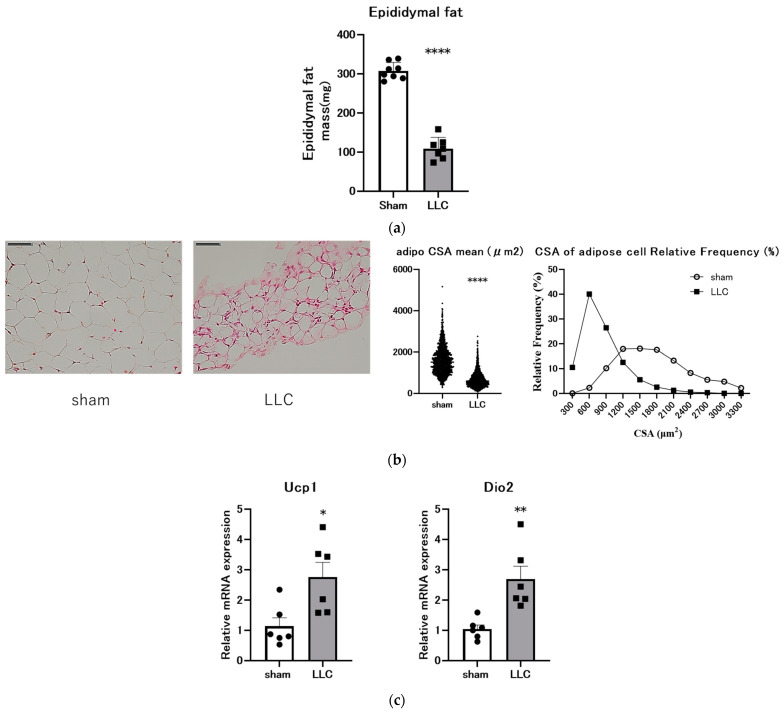
Transplantation of Lewis lung carcinoma (LLC) into the femoral cavity promotes lipolysis. (**a**) Relative change in epididymal fat mass at the endpoint of the experiment (n = 7–8). (**b**) Hematoxylin and eosin-stained sections (Scale bar, 50 μm.) and epididymal fat cross-sectional area (CSA) from the LLC and sham groups (n = 6). (**c**) Relative mRNA expression levels of *Ucp1* and *Dio2* (n = 6). Comparisons were statistically tested with a two-tailed unpaired *t*-test for normally distributed data or Mann–Whitney U test for non-parametric data. Significances are shown as * *p* < 0.05, ** *p* < 0.01, and **** *p* < 0.0001.

eWAT mass and morphology were assessed to investigate changes in adipose tissue. The mice bearing LLC exhibited a significant 67% reduction in eWAT mass compared with the sham control mice (Figure 3a). Consistent with this finding, eWAT adipocyte CSA was markedly decreased in the LLC group (Figure 3b). Moreover, the mRNA expression of the lipolytic genes *Ucp1* [44,45] and *Dio2* [45] was significantly upregulated in the LLC group compared with the sham control group (Figure 3c).

### 3.4. Femoral Implantation of LLC Induces a Systemic Inflammatory Response

**Figure 4:**
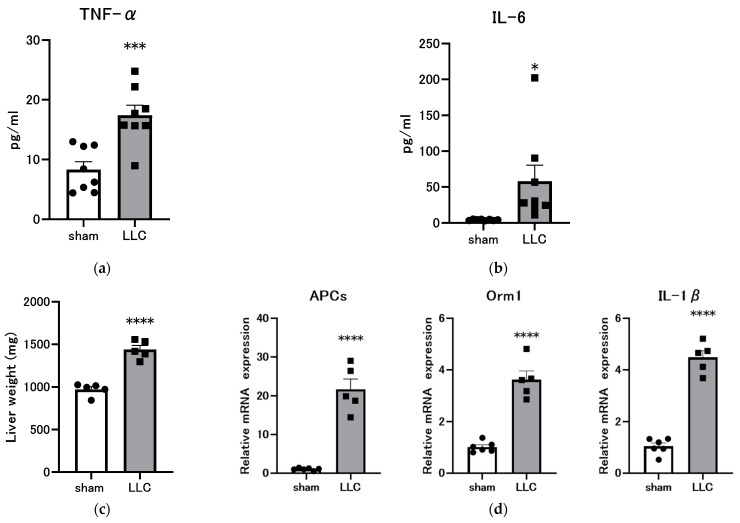
Inflammatory markers in a cancer cachexia model with Lewis lung carcinoma (LLC) implanted in the femoral cavity. (**a**) Plasma concentrations of tumor necrosis factor (TNF)-α and (**b**) interleukin (IL)-6 were measured using a ProQuantum Immunoassay Kit. Data are presented as mean ± SEM (n = 8). (**c**) Comparison of liver and weight (n = 5). (**d**) Comparison of inflammatory markers (*APCs*, *Orm1*, and *IL-1β*) in the liver by quantitative real-time PCR. Data are presented as mean ± SEM (sham n= 6, LLC n= 5). Statistical comparisons were conducted using a two-tailed unpaired *t*-test for normally distributed data or Mann–Whitney U test for non-parametric data. Significance levels are denoted as * *p* < 0.05, *** *p* < 0.001, and **** *p* < 0.0001 compared with the sham group.

Inflammation is a critical component of cancer cachexia, contributing to disease progression. The plasma concentrations of TNF-α and IL-6 were measured to assess inflammatory status. Results revealed that these concentrations significantly increased in the LLC group compared with the sham control group (Figure 4a,b). The liver plays a pivotal role in the pathogenesis of systemic inflammation, responding to acute and chronic inflammatory stimuli. Liver weight was significantly elevated in the LLC group compared with the sham control group (Figure 4c). The inflammatory response plays a role in the pathogenesis of cachexia by contributing to the production of proinflammatory cytokines and acute-phase proteins [46,47]. The present study focused on the acute-phase proteins pentraxin 2/APCS, homologous to human and mouse C-reactive protein, and Orm1, a recently identified appetite suppressant [46,47,48,49]. Hepatic *Apcs* and *Orm1* levels were significantly elevated in the LLC group compared with the sham control group (Figure 4d). Additionally, hepatic *IL-1β* levels were markedly increased in the LLC group compared with the sham control group (Figure 4d).

### 3.5. Behavioral Experiments Were Conducted on Mice with LLC Cells Implanted in the Femoral Bone Marrow Cavity

**Figure 5:**
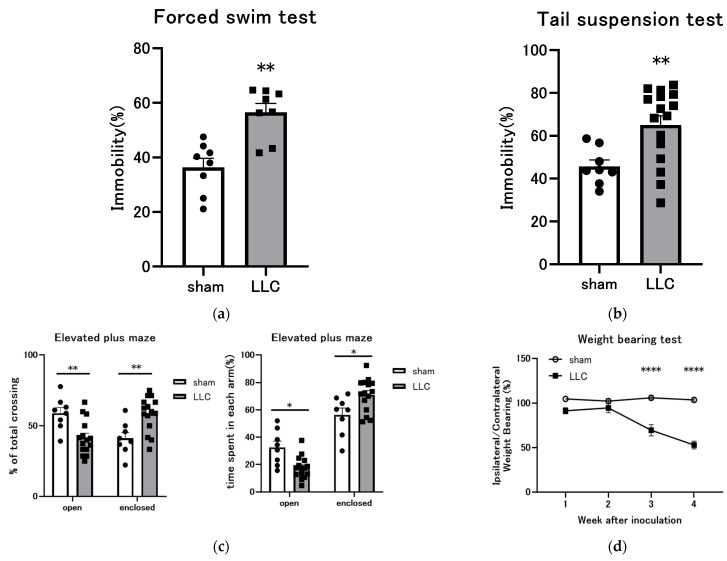
Behavioral evaluation in a cancer cachexia model with Lewis lung carcinoma (LLC) implanted in the femoral cavity. (**a**–**c**) Behavioral tests were performed 4 weeks after LLC transplantation. (**a**) Immobility times were compared between the LLC and sham groups in the forced swim test (FST) 4 weeks post-LLC inoculation (n = 8). (**b**) Immobility time was measured in the tail suspension test (TST) (sham n= 8, LLC n= 16). (**c**) Time spent and entry rate in the open arms of the elevated plus maze (EPM) were recorded (sham n = 8, LLC n = 16). (**d**) Weight-bearing capacity of the hindlimb of mice inoculated with LLC was measured (sham n = 16, LLC n = 14). Statistical comparisons were conducted using a two-tailed unpaired *t*-test for normally distributed data or Mann–Whitney U test for non-parametric data. Significance levels are denoted as * *p* < 0.05, ** *p* < 0.01, and **** *p* < 0.0001 compared with the sham group.

Behavioral experiments were conducted on mice with LLC cells implanted in the femoral bone marrow cavity to assess the development of psychiatric symptoms associated with cachexia. FST and TST are well-established behavioral paradigms in rodents commonly used to assess depression-like behaviors [37,38]. FST and TST results showed that the immobility time was significantly longer in the LLC group than in the sham group (Figure 5a,b). Prolonged immobility time is indicative of an increase in depression-like symptoms. The EPM is a commonly utilized animal test in neuroscience to assess anxiety-related behaviors [39,40]. As shown in Figure 5c, shortened open-arm exploration time and reduced entries into the open arms of the EPM indicate increased anxiety-like behavior. Effects on weight-bearing distribution in the LLC model were analyzed using an incapacitance meter tester. In this test, the assessment of static hindlimb weight-bearing asymmetry serves as an indirect measure of pain. Unilateral femoral intramedullary injection of LLC cells resulted in progressive weight-bearing reduction on the ipsilateral hindlimb. A significant decrease in weight-bearing was observed on the ipsilateral hindlimb 3 weeks post-inoculation in the LLC group compared with the sham group, suggesting increased discomfort associated with limb weight-bearing in the LLC group (Figure 5d).

## 4. Discussion

Recent advancements in understanding the pathophysiology of cancer cachexia have identified potential therapeutic targets, including tumor-derived mediators such as leukemia inhibitory factor (LIF) [50], zinc alpha glycoprotein (ZAG) [51,52], activin A [53], and growth differentiation factor 15 (GDF-15) [54,55]. In addition to targeting tumor-derived factors, anti-inflammatory agents, beta-blockers [14,15,56,57], and compounds such as enobosarm [14,58] and anamorelin [16,17,18,59] have shown promise in preclinical models. However, they have limited clinical applications for mitigating cachexia symptoms. Despite recent advances, current therapeutic strategies have limited efficacy in fully reversing cancer cachexia. This is primarily due to an incomplete understanding of the underlying pathophysiological mechanisms. Preclinical models are essential for elucidating the complexities of cachexia and identifying novel therapeutic targets. Therefore, preclinical research must be intensified to identify compounds with translational potential. In this study, we developed a CIBP model by injecting LLC cells into the intramedullary cavity of the femur and evaluated the manifestation of cachexia-related symptoms in this model.

Compared with the sham operation, transplantation of LLC cells into the femur led to significant weight loss and decreased consumption of food and water (Figure 1a–d). The results demonstrate the significant impact of LLC transplantation to the femoral bone marrow cavity on body weight and food intake, highlighting the importance of this model in studying cachexia-related outcomes. Cancer-related anorexia is a prevalent complication in advanced cancer, affecting a substantial proportion of patients [60,61]. This condition contributes significantly to weight loss and the development of cachexia and sarcopenia [59]. Anorexia in patients with cancer is multifaceted, with contributing factors including nausea, altered taste, constipation, fatigue, pain, and depression [1]. Anorexia and associated weight loss are independently associated with poor prognosis and accelerated disease progression [62]. Moreover, a multi-institutional study has linked weight loss, decreased grip strength, anorexia, and fatigue to diminished quality of life in patients with advanced cancer [63]. In the present study, the LLC group exhibited a temporal progression of symptoms, with pain and decreased grip strength emerging at 3 weeks after transplantation, followed by changes in mental status at 4 weeks after transplantation. These combined factors may have contributed to the decline in food and water consumption after day 21 (Figure 1a,b, Figure 2c, and Figure 5a–d).

Skeletal muscle loss is a key feature of cancer cachexia. Consistent with various animal models of cachexia [22,25,26,27,29] and cancer cachexia in patients with cancer [5,64], the model established in the present study showed a significant reduction in muscle mass (Figure 2a,b). Assessment of sarcopenia by measuring grip strength or skeletal muscle mass is critical for the clinical evaluation of cachexia. Notably, skeletal muscle mass is a prognostic indicator of mortality in cancer cachexia [5,65,66]. Cardiac muscles are also affected by cancer cachexia. Patients with cancer cachexia exhibit reduced cardiac mass and impaired left ventricular systolic function, often presenting with dyspnea, decreased exercise tolerance, and fatigue [67]. Preclinical studies have reported reduced heart size and wall thickness in cachectic animal models, supporting these clinical observations [68,69]. In the present study, the mice in the LLC group exhibited a lower mass of various skeletal muscles and the heart than those in the sham control group (Figure 2a). The quantification of TA myofiber CSA by H&E staining demonstrated that the mean myofiber CSA was diminished in the LLC group relative to the sham group (Figure 2b). Moreover, the muscle mass loss was functionally reflected by decreased grip strength (Figure 2c). Metabolic dysfunction of skeletal muscle represents a significant contributing factor to skeletal atrophy, which is characterized by increased protein degradation [70]. These processes are primarily mediated by the ubiquitin–proteasome and autophagy–lysosome pathways. Upregulation of E3 ubiquitin ligases, including MuRF1 and Atrogin-1, is a hallmark of muscle atrophy [71]. Additionally, autophagy, an intracellular degradation process, is activated in muscle-wasting conditions and is associated with increased expression of autophagy markers such as BNIP3 and LC3B in patients with cancer [42,43]. Examining TA muscles for the expression of muscle atrophy-related genes revealed higher expression levels in the LLC group compared with the sham group (Figure 2d). These findings underscore the complex interplay among muscle mass, grip strength, and muscle atrophy-related gene expression in this model.

In addition to skeletal muscle wasting, adipose tissue atrophy represents a pivotal element of weight loss in cancer cachexia. Browning of white adipose tissue is a systemic feature of cachexia, contributing to increased thermogenic energy expenditure and negative energy balance. Patients with cancer cachexia exhibit a high prevalence of beige adipocytes, and increased brown adipose mass is associated with poor prognosis [71,72]. WAT browning, characterized by increased beige adipocyte number and UCP1 expression, accelerates energy expenditure and exacerbates cachexia progression [44,45]. In the present study, eWAT mass and CSA were significantly decreased in the LLC group compared with the sham group (Figure 3a,b). Furthermore, the mRNA expression of the lipolysis-related genes *Ucp1* and *Dio2* was elevated in the LLC group compared with the sham group (Figure 3c). These results indicate that tumor growth on the femur significantly alters eWAT properties and upregulates lipolysis-related gene expression in this model.

Chronic inflammation is characterized by elevated levels of proinflammatory cytokines. Proinflammatory cytokines, including C-reactive protein (CRP), TNF-α, and IL-6, contribute to muscle wasting and adversely affect patient survival [73,74,75]. Additionally, these proinflammatory cytokines are instrumental in the emergence of secondary nutritional impact symptoms (s-NIS), including early satiety, pain, constipation, depression, and nausea [76]. Femoral bone marrow cavity implantation of LLC cells induced a systemic inflammatory response characterized by elevated circulating levels of inflammatory cytokines, including TNF-α and IL-6 (Figure 4a,b). Hepatomegaly was observed in the LLC-bearing mice (Figure 4c). The liver plays a role in the development of the systemic inflammatory phenotype of cachexia. This is achieved through the production of acute-phase proteins and proinflammatory cytokines [46,47]. We observed significant increases in the levels of acute-phase proteins mRNA (*Apcs* and *Orm1*) and *IL-1β* in the LLC group (Figure 4d). APCS, the murine homolog of human C-reactive protein, is a major acute-phase reactant primarily induced by IL-6 [47,48,77]. Another acute-phase protein, ORM1, is a potential contributor to anorexia through its role in the excessive activation of hypothalamic leptin receptor signaling [48,49]. In the present study, both *Apcs* and *Orm1* were induced, suggesting the activation of systemic inflammatory and anorexia-inducing pathways. Furthermore, hepatic *IL-1β* induction supports the role of the liver as a central inflammatory site in this model.

Patients with cancer cachexia frequently present with a constellation of symptoms that have a detrimental impact on appetite. Nutritional symptoms that may be affected by the disease process include unrelieved pain, early satiety, chronic nausea, anxiety, depression, and asthenia [13,78,79]. Psychological symptoms (s-NIS) have been well characterized in previous studies using the CIBP model [34,35]. Therefore, a series of behavioral experiments were conducted to assess whether these symptoms also occur in this model. TST and FST results showed that the mice with LLC implanted in the femoral bone marrow cavity showed prolonged immobility time, suggesting depression-like behavior (Figure 5a,b). Additionally, the mice exhibited a diminished tendency to explore the open arms of the EPM, which is indicative of elevated anxiety (Figure 5c). Finally, weight-bearing asymmetry was present in the hind limbs of the mice treated with LLC. This result indicates that the pain experienced by the mice altered the weight applied to the limb, resulting in increased discomfort (Figure 5d). The results of these experiments demonstrate that this CIBP model also exhibits complex behavioral phenotypes characterized by depression, anxiety, and pain-like behaviors. Several studies suggest that the presence of pain is associated with depression, reduced energy intake, reduced physical activity, fatigue, and muscle weakness, all of which may contribute to cachexia [80,81,82]. Given the heterogeneous nature of cancer and the multifaceted pathophysiology of cachexia, a comprehensive understanding of the underlying mechanisms may require the evaluation and validation of pathomechanisms in multiple models rather than relying on a standardized paradigm (Table 2). The CIBP model could serve as a novel tool for cachexia research and may help elucidate the mechanisms underlying basic cachexia symptoms, s-NIS, and cachexia symptoms associated with cancer-related pain. Considering the multifactorial nature of cancer cachexia, investigators must understand the characteristics of available models to select the appropriate one for hypothesis formation. In conclusion, the present study has demonstrated whether the CIBP mouse model, which recapitulates s-NIS (pain, depression, and anxiety), exhibits various features of cachexia. The model offers a valuable platform for advancing cancer cachexia research, facilitating a deeper understanding of the etiology of cachexia and the development and evaluation of novel intervention strategies.

## Figures and Tables

**Table 1:** Primer sequences used for quantitative real-time PCR.

Gene	Primer Sequences (Forward and Reverse, 5′-3′)	Accession No.
*G* *apdh*	AGGTCGGTGTGAACGGATTTG TGTAGACCATGTAGTTGAGGTCA	NM_008084
*Atrogin*	CTCCAAGCCAAAGTCCTTAGAG AGGAGCTGTCATTAGGGACATC	NM_026346
*MuRF-1*	TCCTGATGGAAACGCTATGGAG ATTCGCAGCCTGGAAGATGT	NM_001039048
*Bnip3*	CAGAGCGGGGAGGAGAAC GAGGCTGGAACGCTGCTC	NM_009760
*Gabarapl1*	GGACCACCCCTTCGAGTATC CCTCTTATCCAGATCAGGGACC	NM_020590
*Ucp1*	AAGCTGTGCGATGTCCATGT AAGCCACAAACCCTTTGAAAA	NM_009463
*Dio2*	AAGCTGTGCGATGTCCATGT AAGCCACAAACCCTTTGAAAA	NM_010050
*Apcs*	AGACAGACCTCAAGAGGAAAGT AGGTTCGGAAACACAGTGTAAAA	NM_011318
*Interleukin 1β (IL-1β)*	GCAACTGTTCCTGAACTCAACT ATCTTTTGGGGTCCGTCAACT	NM_008361
*Orm1*	CGAGTACAGGCAGGCAATTCA ACCTATTGTTTGAGACTCCCGA	NM_008768

**Table 2:** Characteristics of preclinical cancer cachexia models.

Model	Species	Cell/Induction Method	Injection Site/Tumor Site	Time to Onset of Cachexia (Body Weight Loss)	Report of an Experiment on the Effects of NIS	Cancer Pain Characteristics	Study Limitations	Reference
Lewis Lung Carcinoma CIBP model	Mouse (C57BL/6)	Lewis lung cancer injection (5 × 10^4^ cells)	Femoral bone marrow cavity	3–4 weeks (post-transplantation)	Depression: FST, TST Anxiety: EPM Pain: Weight bearing, von Frey test, cold plate tests	Cancer-induced bone pain (quantifiable).	Rapidly progressive cachexia. Not suitable for long-term studies.	In this study, [35]
Lewis Lung Carcinoma model	Mouse (C57BL/6)	Lewis lung cancer injection (5 × 10^5^~1 × 10^6^ cells)	Subcutaneous (flank) or intramuscular	1–2 weeks (post-transplantation)	Depression: FST, TST	Localized pain may be caused by the tumor compressing the surrounding tissues (not quantifiable).	Rapidly progressive cachexia. Not suitable for long-term studies.	[24,29,83,84]
C26 mouse colon cancer model	Mouse (BALB/c, CD2F1)	Colon-26 injection (5 × 10^5^~1 × 10^6^ cells)	Subcutaneous (flank) or intramuscular	1–3 weeks (post-transplantation)	Depression: FST, TST Anxiety: EPM	Localized pain may be caused by the tumor compressing the surrounding tissues (not quantifiable).	Rapidly progressive cachexia. Not suitable for long-term studies.	[22,29,83,84]
B16F10 mouse melanoma model	Mouse (C57BL/6)	B16F10 injection (5 × 10^4^~1 × 10^6^ cells)	Subcutaneous	1–3 weeks (post-transplantation)	Fatigue: Total locomotion activity	Localized pain may be caused by the tumor compressing the surrounding tissues (not quantifiable).	Rapidly progressive cachexia. Not suitable for long-term studies.	[24,84]
85As2 human gastric cancer model	Immunodeficient animals (BALB/c nu/nu mice, F344/NJcl-rnu/rnu rats)	85As2 injection (1 × 10^6^~1 × 10^7^ cells)	Subcutaneous	1–2 weeks (post-transplantation)	No reports	Potential for localized pain due to tumor compression of surrounding tissues (not quantifiable).	Rapidly progressive cachexia. Not suitable for long-term studies.	[25,85]
Yoshida AH-130 rat hepatoma cell model	Rat (Wistar)	Yoshida AH-130 injection (1 × 10^8^ cells)	Intraperitoneal	1–2 weeks (post-transplantation)	Depression: FST	Potential for localized pain due to tumor compression of surrounding tissues (not quantifiable).	Rapidly progressive cachexia. Not suitable for long-term studies.	[26,83]
Apc Min/+ mouse model	Mouse (Genetic mutation)	Spontaneous mutation in the Apc gene	Intestinal tract (mainly colon)	12–20 weeks of age	Fatigue: Total locomotion activity	Minimal direct pain: however, tumor burden can cause discomfort and a decrease in activity (not quantifiable).	Prenatal genetic modification may affect development.	[27,86,87,88]
Inhibin alpha subunit knockout model	Mouse (Genetically modified)	Genetic modification (Inhhibin α (-/-))	Gonadal and adrenal	6–12 weeks of age	No reports	Minimal direct pain: however, tumor burden can cause discomfort and a decrease in activity (not quantifiable).	Prenatal genetic modification may affect development.	[28,89,90,91]
KPP model	Mouse (Genetically modified)	Genetic modification (Ptf1a^Cre-ERTM^; Kras^LSL-G12D^; Pten-^flox^)	Pancreas	Between 75 and 90 days after tamoxifen administration. (Initiated with tamoxifen between 24 and 28 days (Tumor formation). Median survival 3.5 months)	Fatigue: Total locomotion activity	Pancreatic tumor enlargement, nerve invasion, and local tissue compression may cause pain (not quantifiable).	Lose normal pancreatic parenchyma.	[29]

## Data Availability

All study data are included in the main text.
